# Innovative, sustainable, and circular agricultural systems for the future

**DOI:** 10.1007/s13165-021-00356-0

**Published:** 2021-05-06

**Authors:** Gerold Rahmann, Khalid Azim, Irena Brányiková, Mahesh Chander, Wahyudi David, Jan Willem Erisman, Daniel Grimm, Andrew Hammermeister, Li Ji, Anja Kuenz, Anne-Kristin Løes, Wan Abd Al Qadr Imad Wan-Mohtar, Daniel Neuhoff, Saliou Niassy, Victor Olowe, Mia Schoeber, Jessica Shade, Jörg Ullmann, Arnold van Huis

**Affiliations:** 1 ISOFAR, c/o Thünen-Institute, Trenthorst 32, 23847 Westerau, Germany; 2 UR-PIC, Institut National de la Recherche Agronomique (INRA), CRRA Agadir, Agadir, Morocco; 3 Institute of Chemical Process Fundamentals of the CAS, Prague, Czech Republic; 4 Indian Veterinary Research Institute, Bareilly, India; 5 Universitas Bakrie, South Jakarta, Indonesia; 6 Leiden University, Leiden, The Netherlands; 7 Thünen-Institute, Brunswick, Germany; 8 Dalhousie University, Halifax, Canada; 9 China Agricultural University, Beijing, China; 10 Norwegian Center for Organic Agriculture (NORSØK), Tingvoll, Norway; 11 Institute of Biological Sciences, Faculty of Science, Universiti Malaya, Kuala Lumpur, Malaysia; 12 University of Bonn, Bonn, Germany; 13 International Centre of Insect Physiology and Ecology (icipe), Nairobi, Kenya; 14 Federal University of Agriculture, Abeokuta, Nigeria; 15 University of Natural Resources and Life Science, Austria and GIZ, Addis Ababa, Ethiopia; 16 The Organic Center, Washington, DC USA; 17 Roquette Klötze GmbH & Co. KG, Klötze, Germany; 18 Wageningen University, Wageningen, The Netherlands

**Keywords:** Food security, Circular chains of nutrients, Sustainable food production, Organic agriculture, Landless food

## Abstract

This special issue presents the outcomes from “*Designing sustainable and circular agricultural systems for the year 2100*,” the joint scientific workshop of ISOFAR, the Thünen-Institute, and INRA-Morocco, which was held from November 14 to 16, 2019 in Marrakesh, Morocco. Nineteen scientists from a broad array of background and nationalities came together with the understanding that food security globally is at risk, especially in the post-2050 timeframe. Current concepts, strategies, measures, and scientific efforts carried out by governments, NGOs, businesses, and societies do not deliver satisfying solutions for how to sustainably produce enough healthy and affordable food to support the global population. With the economic and social impact of the Covid-19 pandemic in 2020, it became even more evident that food security is a challenge. This workshop took an innovative approach to addressing the challenges of future agriculture by considering sustainable, circular agricultural systems. Participants presented research results on algae-based food, edible insects, mushrooms, novel concepts for nutrient management, bioreactor-based farming, sustainable food culture, as well as sensor- and remote-controlled automatic food production. This special issue presents the papers contributed to the workshop and the results of the discussions.

## The problem of food security in 2100

Food security**—**measured by the availability of food and individuals’ ability to access it—was, is, and will be a permanent challenge for humankind. On the one hand, an increase of the global human population from 7.6 in 2017 toward 11.2 billion people in the year 2100 (UN 2017; Rahmann et al. 2017) requires an intensification of food production. Land-use changes, like deforestation and conversion of natural grasslands into cropland ( “Land Use, Land-Use Change and Forestry,” LULUCF; see UNFCC 2021), are not a feasible solution because they are environmentally catastrophic and limited by the small amount of natural landscapes still available on earth. On the other hand, the structure of food production today is not sustainable enough and requires rethinking. A major challenge is the global decreasing of soil fertility, in addition to rising water scarcity and environmental contamination (DeLong et al. 2015). Fertilizers and pesticides improve food security, especially in the short term, but often endanger farm-related biodiversity, decrease soil quality, and lead to health risks for the consumer (Foley et al. 2005). Finally, climate change will have a significant, potentially destabilizing impact on critical crops (Rahmann et al. 2017). All food systems need to change toward intensification and sustainability, with an emphasis on circular economic models. The United Nations point out these challenges in the Sustainable Development Goals (e.g., SDG 2, UN 2015), and all nations have pledged to contribute. From a scientific point of view, it is possible to achieve these goals, but creative thinking is required to address these multifaceted issues (FAO 2019; FAO 2021a).

These challenges are especially problematic for continents such as Africa, where the food security is already a challenge, with population increase predicted to reach a level above which the natural resources for food production (including fisheries) will not be sufficient to solve the growing demand. Increased reliance on imports are not a stable solution because it is unclear whether these nations will have the financial capabilities and infrastructural capacities in rural areas for this option. Food sovereignty for Africa will be almost impossible to achieve, if the population grows from 1.3 (year 2017) toward 3.6 or even 4.5 billion people in 2100 (UN 2017; Hoornweg and Pope 2017; Rahmann et al. 2020), especially if food production does not rapidly improve toward the high yielding levels of developed agricultural countries. Today, 260 of the 820 million undernourished people in the world live in Africa, and resources are already scarce (FAO 2019). Continental and regional difficulties for food security does include limited farmland, water scarcity, under-developed food chain infrastructure, lack of food-related education, changing climate with less predictable weather, lack of capital, poverty, conflict, unstable markets, and limitations in farm inputs (WHO 2018).

Despite the concentrated effort over the last few decades, there is no scientific evidence that an end to hunger and malnutrition is in sight (Rahmann and Grimm 2020). There are many scientific efforts and promises made by development projects and policy papers attempting to tackle the African challenges (FAO 2021b). Approaches are scaling-up of (a) high external input–high output, mechanized agriculture, (b) agro-ecological measures, (c) genetically modified, pest- and drought-resistant crops on arid land, (d) import of food (trade or aid), and/or (e) changing habits and diets of people. However, none of these, even in combination, may be enough to increase food production faster than population growth. Sustainability, though an often-invoked concept, is likely to end up as a side-note in these upscaling and intensification efforts.

There is, however, no shortage of innovative ideas for the future of food production—be it vertical farming, agroforestry, aquaculture, or synthetic food (Rahmann et al. 2017). The problem is that no single solution will suffice, and no coherent model integrating all these approaches has yet been accepted as a guideline for future research and development. This is what the participants of the workshop described in this paper set out to do: to bring together different ideas and research topics in the context of a circular economic model and to discuss how such a multifaceted model can be used as a template for the global food system in the year 2100.

## The “Designing sustainable and circular agricultural systems for the year 2100” workshop

The starting point to design joint technology concepts is to overcome ideological barriers and disciplinary boundaries. With an “out of the box” approach, solutions are thinkable, even in the context of the worst-case predictions of population growth and degradation of natural resources.

**Fig. 1:**
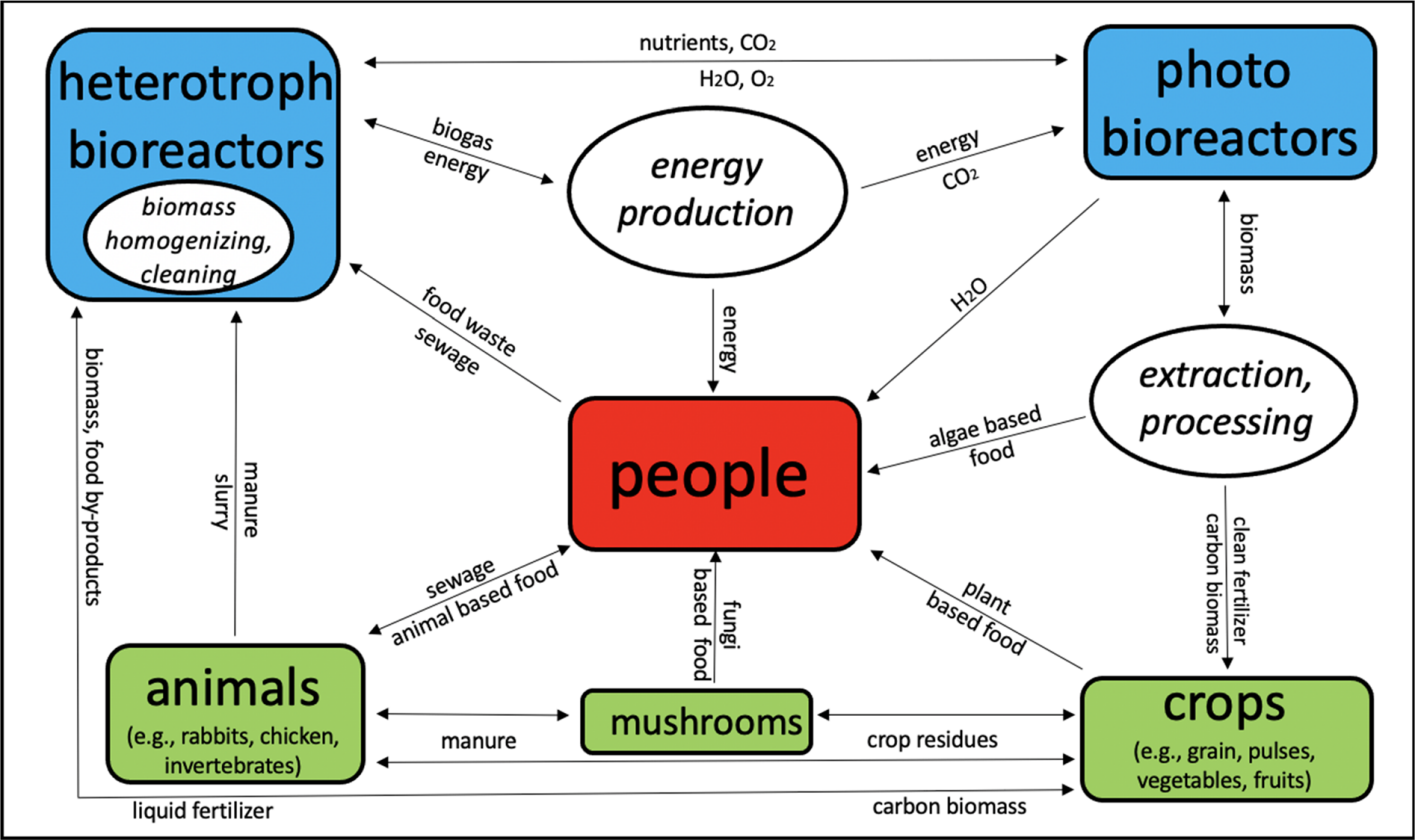
A comprehensive circular and sustainable food chain model (Rahmann et al. 2020)

From November 14 to 16, 2019, in Marrakesh, Morocco, 19 scientists from all over the world representing a wide range of research disciplines exchanged and discussed their ideas for designing sustainable and circular agricultural systems for the year 2100. The conceptual model of a comprehensive circular and sustainable food chain was used to structure the sessions and discussions (Fig. 1).

The model is based on the scientific concept from Johann Heinrich von Thünen (1842): The *Thünen Circles*. The core concept is that the available space for food production is extremely limited (e.g., for Africa in the year 2100, only about 450 m^2^ cropland per person, if the population increases to 5.8 billion). The challenge is thus to produce enough food despite this limitation in a circular and sustainable fashion, without import of food/feed. This is only possible if the available biomass is used very efficiently. Optimizations are possible using traditional food production methods (farming/fishery-based; green chains in the model), but this will not produce sufficient food for the growing population. To increase the efficiency of the system, biomass which is not edible/digestible (by humans/livestock) can be used for off-farm food, energy and fertilizer production (reactor-based; “blue chain” or mushroom cultivation in the “green chain,” Fig. 1).

The structure of the workshop included an “out of the box” approach in a protected environment (“anyone can say anything”), which stimulated creativity to go further with the next steps to solve the future food security problems.

## Suggested contributions for solutions for food security

The most important strategy for increasing food production is to boost yields per hectare. In developed countries successful examples exist, and efforts to replicate these advancements have been attempted with varying success (Neuhoff and Kwesiga 2021; Rahmann et al. 2017). Unfortunately, the knowledge, skills, and resources are not always easily transferable to developing areas. One of the main hurdles is to optimize use of nutrients, especially nitrogen and phosphate, to increase nutrient use efficiency and reduce nutrient pollution. Shade et al. (2020) provided a snapshot of nutrient efficiency in the USA, showing that “*conventional production relies heavily on the creation of new reactive nitrogen whereas organic production primarily utilizes already existing reactive nitrogen.*” Erisman (2020) confirmed the “*potential of a nature-based food system in relation to health and wellbeing*,” as already integrated into practices in regions of the Netherlands.

However, organic agriculture is not always as productive in terms of yields as conventional food production—a critical argument under the extreme limitations of cropland. Neuhoff and Kwesiga (2021) outlines an approach on how to improve soil fertility with “*good farming practices*” and how this can be achieved by small-scale farmers in Africa, with severe resource limitations. The participants confirmed that his proposal of a “*para-organic approach, which integrates classical organic management practices and reasonable use of chemical inputs*” could be a way for Africa to maximize the benefits from both organic and conventional practices (Rahmann et al. 2017). Olowe (2020) provided a Nigerian perspective (most populous country in Africa) by appraising the status of agriculture in the country and articulating strategies to attain sustainable food and nutrition security in the nearest future. Tabrika et al. (2020) used phosphate sludge, a by-product of phosphate mining, in Morocco. Their results “*confirm that composting could be a promising approach for application of PS [phosphate sludge] for P recovery to arable soils.*”

Nevertheless, all such efforts to increase production are likely to be “eaten up” by the increasingly rapid population growth. Therefore, no matter how large the advancements in yields, increasing production on cropland will not suffice as a solution.

If biomass is non-edible/digestible and cannot be used as feed for livestock, another option is the efficient utilization using bioreactors. Undeniably, the easiest way to use food waste is as feed for livestock. Chander and Kannadhasan (2020) give “*an overview of the potential of fruit and vegetable wastes as animal and poultry feed*” in India. If the biomass is not edible for livestock (sewage, manure, sludge), it can be used in biogas reactors to produce renewable energy, and liquid organic fertilizer. Schoeber et al. (2020a, 2020b) have given the example of using biogas reactors for digestion of manure from livestock and sewage from humans in small scale and rural households in Ethiopia. They show that it is feasible, even on small-scale farms in remote rural areas, to set up systems in which “*organic waste is transformed to biogas utilized for light and cooking, and slurry, a nutritious organic fertilizer and source of organic matter.*” Løes (2020) from Norway showed, that “*animal bones, and precipitated struvite from waste-water, are examples of materials which may be applicable in bioreactors*” and Kuenz et al. (2020), that “*wastewater treatment as a means of nutrient recycling will be one of the most important tasks in the future.*” To this end, not only the heterotrophic bioreactors, which are currently used for this purpose, but also autotrophic photobioreactors show great potential, especially if these two reactor types would be combined.

Innovative food and feed sources can come from reactors. Branyikova and Lucakova (2020) explained why “*microalgae can be regarded as ‘microplants,’ able to convert carbon dioxide and water into organic compounds via photosynthesis. Nevertheless, comparing to higher plants (agricultural crops) the microalgae have much higher areal [farm land space] productivities; high content of proteins, vitamins, antioxidants, polyunsaturated fatty acids and other health-promoting components. Moreover, they can be produced in non-arable areas requiring low-cost inputs.*” Ullmann and Grimm (2021) concluded that “*the cultivation of algae can make an important contribution to the food security of future generations, especially in regions of the world where, due to population growth, the available cropland is likely to be insufficient*.” Last but not least, the sustainability of bioreactors can be high, as they do not need pesticide or antibiotic usage, and nutrients are efficiently converted into biomass. Creating a novel algae industry could have an important impact on the local economies, creating new products, new value chains, and jobs.

Mushrooms, insects, or microalgae are other potential high-value food products, which can be produced from non-edible/not digestible biomass or sewage or slurry. Van Huis (2020) from the Netherlands brought insects into the discussion. He writes that “*insects can transform low value organic side streams into high value protein products*” and that “*the insect sector is maturing fast, but still faces many challenges, which can only be met when all stakeholders cooperate closely.*” In a review, Grimm et al. (2020) explained that “*edible mushrooms are cultivated mainly on non-edible, ligno-cellulosic plant materials, thereby turning agricultural wastes to high quality products.*” While mushrooms have a high potential to produce food which is particularly well suited as a meat substitute, they can also facilitate the production of high-quality compost. Another new area of mushroom cultivation that shows great potential is the production of high-protein animal feed in bioreactors, as Wan-Mohtar et al. (2020) justified in experiments with Tilapia fish: “*an alternative to ineffective cash crops practices that destroy arable land.*” This shows, that not only one solution is available, but several and they can be combined to find even better synergies.

## Conclusion

While producing enough food to feed the population of 2100 is possible, some countries, especially those in Africa, are likely to face severe problems with food security. Unfortunately, there is a high likelihood that billions of people in Africa and other developing areas will face hunger and/or malnutrition. The tragic consequences (famines, stunted youth, poverty, migration, etc.) will not only burden these countries but also the world as a whole. Global trade and/or food aid is not a sustainable long-term solution, as food sovereignty with plentiful healthy and affordable food must be the target of any country. The question remains: how can this be achieved? Simple “one size fits all” protocols will not provide solutions across the globe. All possibilities to improve and increase food production must be considered comprehensibly and sustainably, and to achieve this we will need increased discussions and participants, including science.

This workshop showcased several options to increase yields per hectare, use novel food sources, improve food waste and nutrient management cycles, and produce nutrients off-farm. Most of the disruptive and innovative food research is done in developed countries, who do not face the extreme challenges of Africa and parts of Asia, and many solutions are not designed and/or tested under conditions with socio-economic and agro-ecological deficits. Adaptation toward robust, cheap, and easy-to-use technology is necessary, and explorations of these potential solutions must include collaborations within target countries.

In addition, the participants identified the problem that many solutions are invented and developed in isolation and under specialized scientific disciplines, but not merged into an interdisciplinary conceptional frame (e.g., see Fig. 1). They suggested that future efforts should include more cooperation and less competition between scientists and disciplines, as well as more “down to earth” research with convincing, effective, and practical solutions under difficult conditions. Participatory research structures, together with other disciplines, practitioners, and the public, including citizen science, would avoid inefficient resource allocation and allow faster implementation of successful results under specific local conditions (learning from each other).

A cooperative, holistic, and “real impact” research model to reduce food security risks and sustainably increase food sovereignty needs a creative experimental playground for scientists, business and decision-makers and, last but not least, the target audience (e.g., farmers and consumers). To establish experimental facilities in or adjacent to a metropolis in Africa or India would be an excellent opportunity to understand, develop, and prove such innovative solutions as are outlined in Rahmann et al. (2020). A follow-up workshop should be used to design such a facility and infrastructure of “LandLessFood,” together with other disciplines, stakeholders, and decision-makers.
